# The genome sequences of the male and female green-veined white, *Pieris napi* (Linnaeus, 1758)

**DOI:** 10.12688/wellcomeopenres.17277.1

**Published:** 2021-10-26

**Authors:** Konrad Lohse, Alex Hayward, Sam Ebdon, Darwin Tree of Life Barcoding collective, Wellcome Sanger Institute Tree of Life programme, Wellcome Sanger Institute Scientific Operations: DNA Pipelines collective, Tree of Life Core Informatics collective, Darwin Tree of Life Consortium

**Affiliations:** 1 Institute of Evolutionary Biology, University of Edinburgh, Edinburgh, UK; 2 University of Exeter, Penryn, UK

**Keywords:** Pieris napi, green-veined white, genome sequence, chromosomal

## Abstract

We present genome assemblies from a male and female *Pieris napi *(the green-veined white; Arthropoda; Insecta; Lepidoptera; Pieridae). The genome sequences of the male and female are 320 and 319 megabases in span, respectively. The majority of the assembly (99.79% of the male assembly, 99.88% of the female) is scaffolded into 24 autosomal pseudomolecules, with the Z sex chromosome assembled for the male and Z and W chromosomes assembled for the female. Gene annotation of the male assembly on Ensembl has identified 13,221 protein coding genes.

## Species taxonomy

Eukaryota; Metazoa; Ecdysozoa; Arthropoda; Hexapoda; Insecta; Pterygota; Neoptera; Endopterygota; Lepidoptera; Glossata; Ditrysia; Papilionoidea; Pieridae; Pierinae; Pieris; *Pieris napi* (Linnaeus, 1758) (NCBI:txid78633).

## Introduction

*Pieris napi,* green-veined white, is a small circumboreal butterfly that is widespread throughout the British Isles apart from Shetland and parts of the Scottish highlands. Adults can be found laying eggs on wild brassicas over several generations from spring to the beginning of autumn. *P. napi* has seen recent increases in abundance in the UK ( Fox *et al.*, 2015) and is listed as Least Concern in the IUCN Red List (Europe) ( van Swaay *et al.*, 2009). This species has been used to investigate evolutionary dynamics in insect immune system genes, which were shown to harbour elevated genetic diversity and signals of either balancing or positive selection ( Keehnen *et al.*, 2018). *P. napi* has 25 pairs of chromosomes, a genome size of 349.8 Mb ( Hill *et al.*, 2019), and is female heterogametic (WZ). We note the recent production of a high-quality genome assembly for *P. napi* ( Hill *et al.*, 2019), and believe the sequence described here, generated as part of the Darwin Tree of Life project, will further aid understanding of the biology and ecology of this butterfly. Both male and female assemblies were produced to enable correct identification of and discrimination between the sex chromosomes.

## Genome sequence report

**Figure 1.**
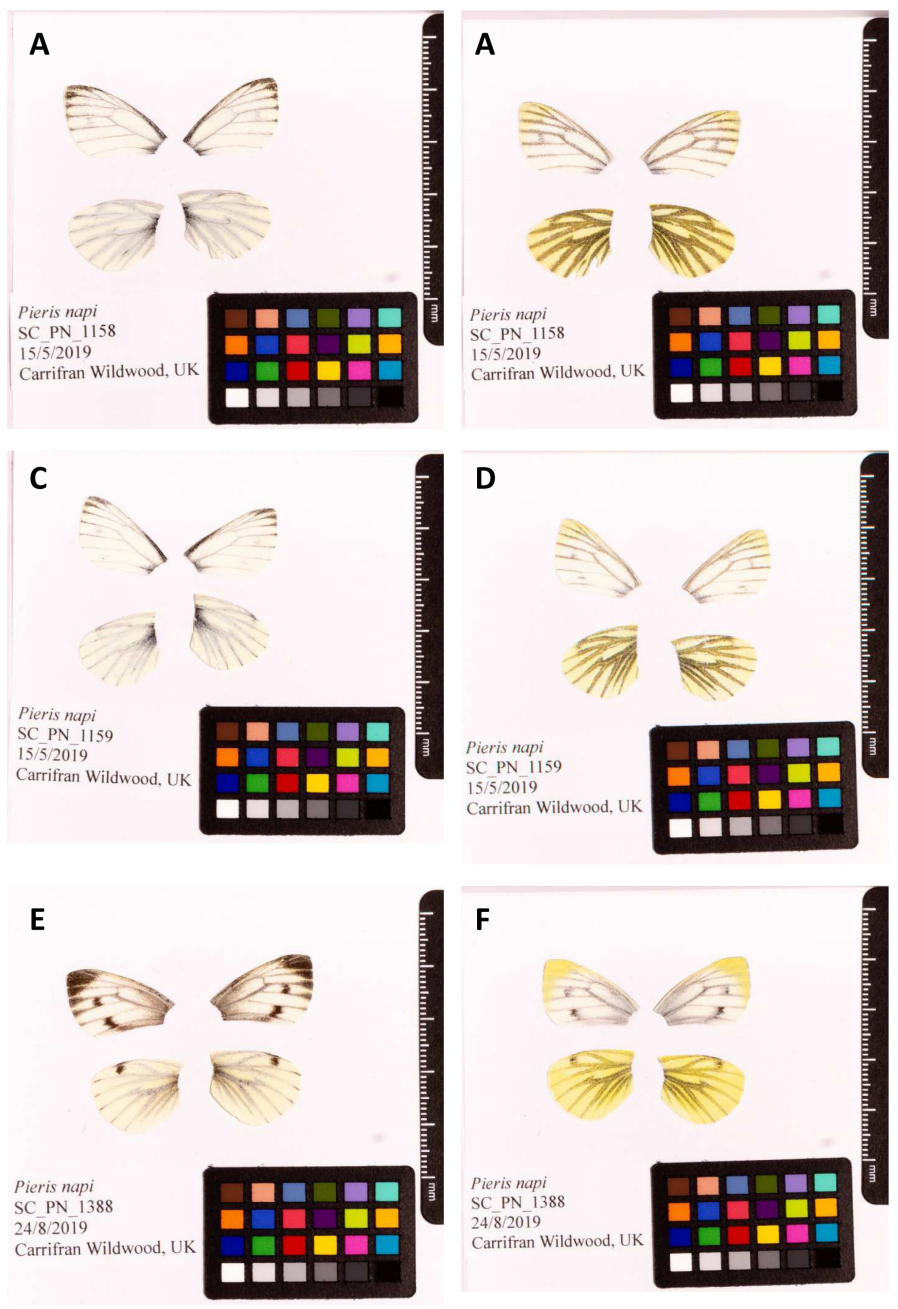
Fore and hind wings of *Pieris napi* specimens from which the genome was sequenced. ( **A**) Dorsal surface view of wings from specimen SC_PN_1158 (ilPieNapi4, male) used to generate Pacific Biosciences and 10X genomics data. ( **B**) Ventral surface view of wings from specimen SC_PN_1158 (ilPieNapi4) used to generate Pacific Biosciences and 10X genomics data. ( **C**) Dorsal surface view of wings from specimen SC_PN_1159 (ilPieNapi1) used to generate Hi-C data. ( **D**) Ventral surface view of wings from specimen SC_PN_1159 (ilPieNapi5) used to generate HiC data. ( **E**) Dorsal surface view of wings from specimen SC_PN_1388 (ilPieNapi1) used to generate Pacific Biosciences and 10X genomics data. ( **F**) Ventral surface view of wings from specimen SC_PN_1388 (ilPieNapi1, female) used to generate Pacific Biosciences and 10X genomics data. All three samples were collected from Carrifran Wildwood, Scotland, UK.

The genomes were sequenced from a single male *P. napi*, ilPieNapi4, and single female, ilPieNapi1, collected from Carrifran Wildwood, Scotland (latitude 55.400132, longitude -3.3352) ( Figure 1). Hi-C data for both assemblies were generated from a second male *P. napi*, ilPieNapi5, collected from the same location ( Figure 1). A total of 91-fold coverage in Pacific Biosciences single-molecule long reads and 107-fold coverage in 10X Genomics read clouds were generated for the male assembly; 60- and 52-fold coverage were generated using the Pacific Biosciences and 10X Genomics technologies for the female assembly. Primary assembly contigs were scaffolded with chromosome conformation Hi-C data. Manual assembly curation of the male assembly corrected 33 missing/misjoins and removed seven haplotypic duplications, reducing the assembly size by 0.71% and scaffold number by 25.00%, and increasing the scaffold N50 by 3.75%. Manual assembly curation of the female assembly corrected 105 missing/misjoins and removed 28 haplotypic duplications, reducing the assembly size by 1.22% and scaffold number by 27.59%, and increasing the scaffold N50 by 1.98%.

**Figure 2.**
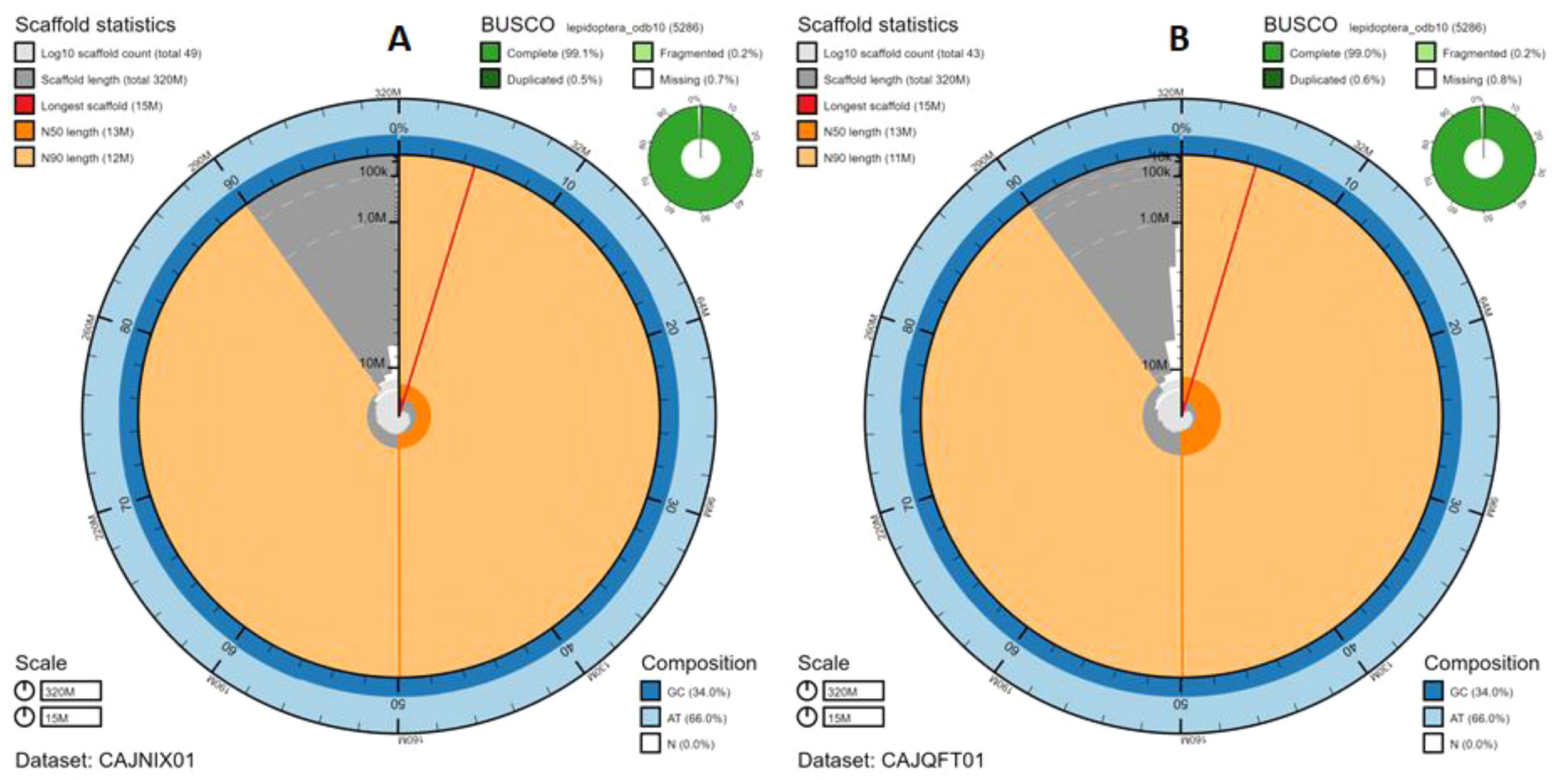
Genome assembly of *Pieris napi*, ilPieNapi4.1 (male, A) and ilPieNapi1.1 (female, B): metrics. The BlobToolKit Snailplot shows N50 metrics and BUSCO gene completeness. The main plot is divided into 1,000 size-ordered bins around the circumference with each bin representing 0.1% of the 320,004,350 bp (male, **A**) and 319,202,574 bp (female, **B**) assemblies. The distribution of chromosome lengths is shown in dark grey with the plot radius scaled to the longest chromosome present in the assembly (15,058,180 bp (male) and 14,821,532 bp (female), shown in red). Orange and pale-orange arcs show the N50 and N90 chromosome lengths (12,982,002 and 11,574,744 bp, male; 13,068,865 and 10,703,985 bp, female), respectively. The pale grey spiral shows the cumulative chromosome count on a log scale with white scale lines showing successive orders of magnitude. The blue and pale-blue area around the outside of the plot shows the distribution of GC, AT and N percentages in the same bins as the inner plot. A summary of complete, fragmented, duplicated and missing BUSCO genes in the lepidoptera_odb10 set is shown in the top right. Interactive versions of this figure are available at https://blobtoolkit.genomehubs.org/view/ilPieNapi4.1/dataset/CAJNIX01/snail (male) and https://blobtoolkit.genomehubs.org/view/ilPieNapi1.1/dataset/CAJQFT01/snail (female).

The final male assembly has a total length of 320 Mb in 49 sequence scaffolds with a scaffold N50 of 13 Mb; the final female assembly has a total length of 319 Mb in 43 sequence scaffolds with a scaffold N50 of 13 Mb ( Table 1). Of the male assembly sequence, 99.79% was assigned to 25 chromosomal-level scaffolds, representing 24 autosomes (numbered by synteny to the female assembly), and the Z sex chromosome; of the female assembly sequence, 99.88% was assigned to 26 chromosomal-level scaffolds, representing 24 autosomes (numbered by sequence length) and the W and Z chromosomes ( Figure 2– Figure 5; Table 2). The assemblies have a BUSCO ( Simão *et al.*, 2015) v5.1.2 completeness of 99.1% (single 98.5%, duplicated 0.5%, fragmented 0.2%, missing 0.7%; male) and 99.0% (single 98.4%, duplicated 0.6%, fragmented 0.2%, missing 0.8%; female) using the lepidoptera_odb10 reference set. While not fully phased, the assemblies deposited are of one haplotype. Contigs corresponding to the second haplotype for each assembly have also been deposited.

**Table 1.** Genome data for *Pieris nap* *i*, ilPieNapi4.1 (male) and ilPieNapi1.1 (female).

	Male assembly	Female assembly
*Project accession data*		
Assembly identifier	ilPieNapi4.1	ilPieNapi1.1
Species	*Pieris napi*	
Specimen	ilPieNapi4 (genome assembly); ilPieNapi5 (Hi-C); ilPieNapi6 (RNA-Seq)	ilPieNapi1 (genome assembly); ilPieNapi5 (Hi-C); ilPieNapi9 (RNA-Seq)
NCBI taxonomy ID	NCBI:txid78633	
BioProject	PRJEB43012	
BioSample ID	SAMEA7523140	SAMEA7523287
Isolate information	Male, whole organisms	Female, whole organism (genome assembly); male, whole organism (Hi-C)
*Raw data accessions*		
PacificBiosciences SEQUEL II	ERR6594499	ERR6594498
10X Genomics Illumina	ERR6054471-ERR6054474	ERR6054475-ERR6054478
Hi-C Illumina	ERR6054479	
Illumina PolyA RNAseq	ERR6363261	ERR6787421
*Genome assembly*		
Assembly accession	GCA_905231885.1	GCA_905475465.1
*Accession of alternate haplotype*	GCA_905231895.1	GCA_905475415.1
Span (Mb)	320	319
Number of contigs	76	84
Contig N50 length (Mb)	11	8
Number of scaffolds	49	43
Scaffold N50 length (Mb)	13	13
Longest scaffold (Mb)	15	15
BUSCO * genome score	C:99.1%[S:98.5%,D:0.5%],F:0.2%,M:0.7%,n:5286	C:99.0%[S:98.4%,D:0.6%],F:0.2%,M:0.8%,n:5286
*Gene annotation*		
Number of protein coding genes	13,221	-
Average coding sequence length (bp)	1,720	-
Average number of exons per transcript	10	-
Average exon size (bp)	360	-
Average intron size (bp)	1,929	-

*BUSCO scores based on the lepidoptera_odb10 BUSCO set using v5.1.2. C= complete [S= single copy, D=duplicated], F=fragmented, M=missing, n=number of orthologues in comparison. Full sets of BUSCO scores are available at https://blobtoolkit.genomehubs.org/view/ilPieNapi4.1/dataset/CAJNIX01/busco (male) and https://blobtoolkit.genomehubs.org/view/ilPieNapi1.1/dataset/CAJQFT01/busco (female).

**Figure 3.**
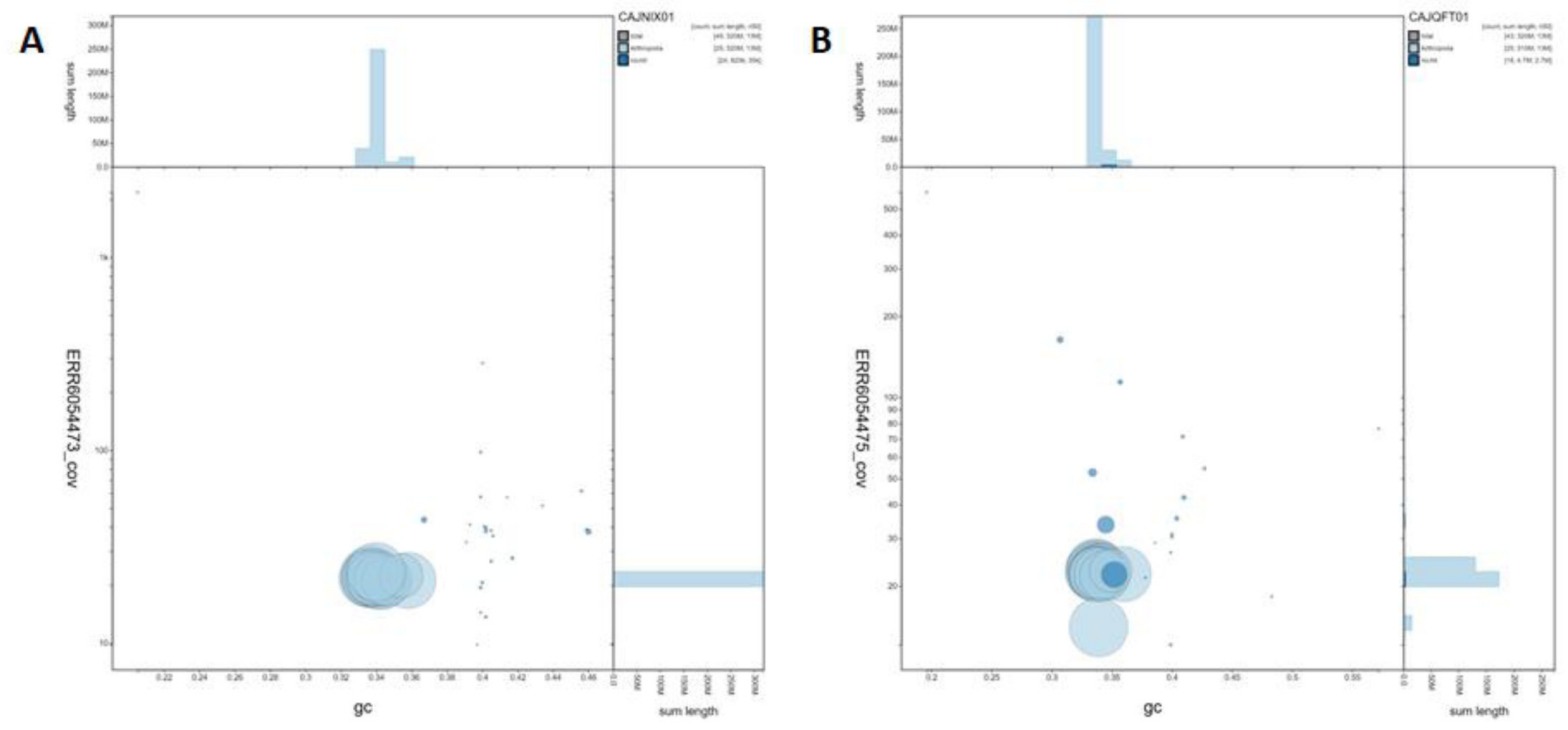
Genome assembly of *Pieris napi*, ilPieNapi4.1 (male, A) and ilPieNapi1.1 (female, B): GC coverage. BlobToolKit GC-coverage plot. Scaffolds are coloured by phylum. Circles are sized in proportion to scaffold length. Histograms show the distribution of scaffold length sum along each axis. Interactive versions of this figure are available at https://blobtoolkit.genomehubs.org/view/ilPieNapi4.1/dataset/CAJNIX01/blob (male, **A**) and https://blobtoolkit.genomehubs.org/view/ilPieNapi1.1/dataset/CAJQFT01/blob (female, **B**).

**Figure 4.**
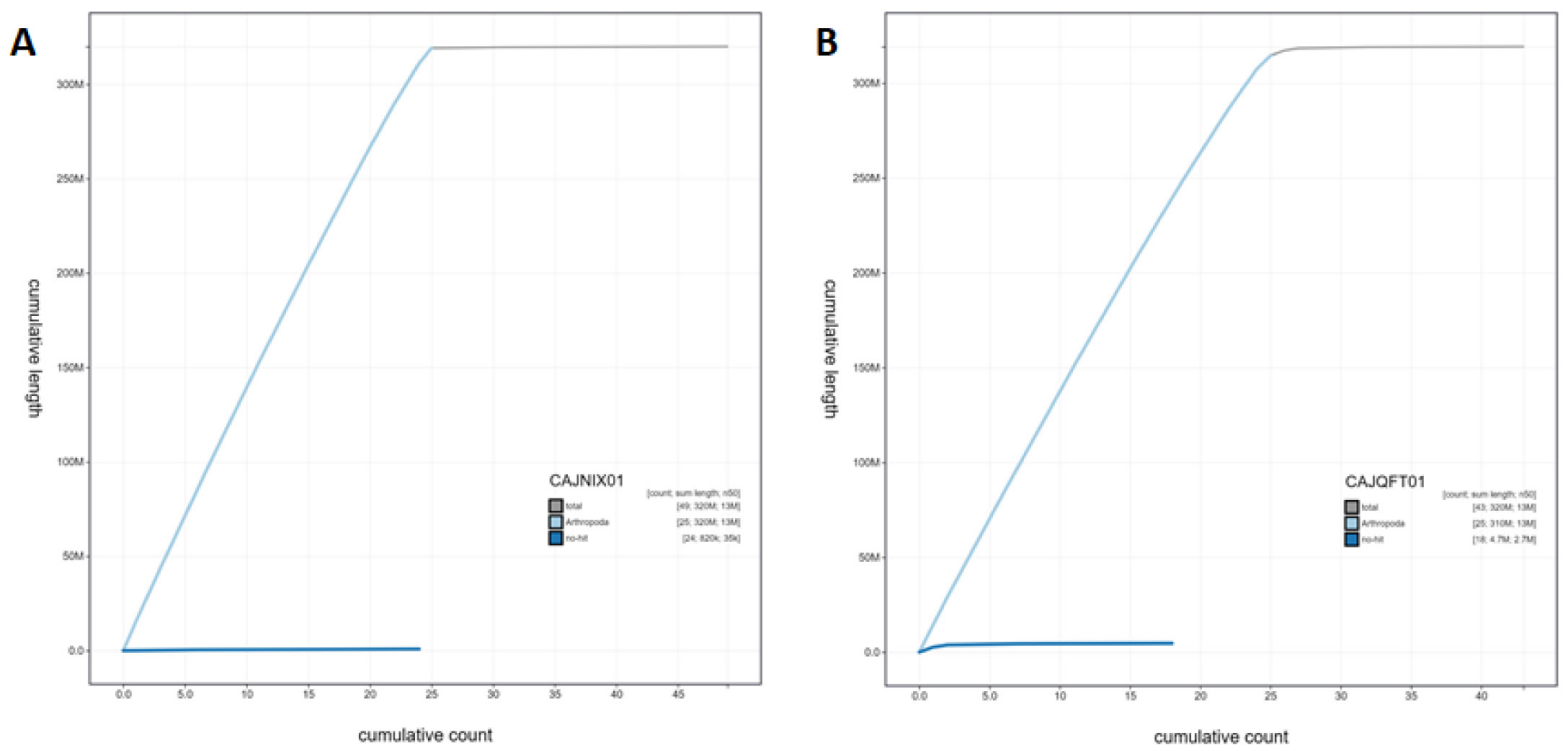
Genome assembly of *Pieris napi*, ilPieNapi4.1 (male, A) and ilPieNapi1.1 (female, B): cumulative sequence. BlobToolKit cumulative sequence plot. The grey line shows cumulative length for all scaffolds. Coloured lines show cumulative lengths of scaffolds assigned to each phylum using the buscogenes taxrule. Interactive versions of this figure are available at https://blobtoolkit.genomehubs.org/view/ilPieNapi4.1/dataset/CAJNIX01/cumulative (male, **A**) and https://blobtoolkit.genomehubs.org/view/ilPieNapi1.1/dataset/CAJQFT01/cumulative (female, **B**).

**Figure 5.**
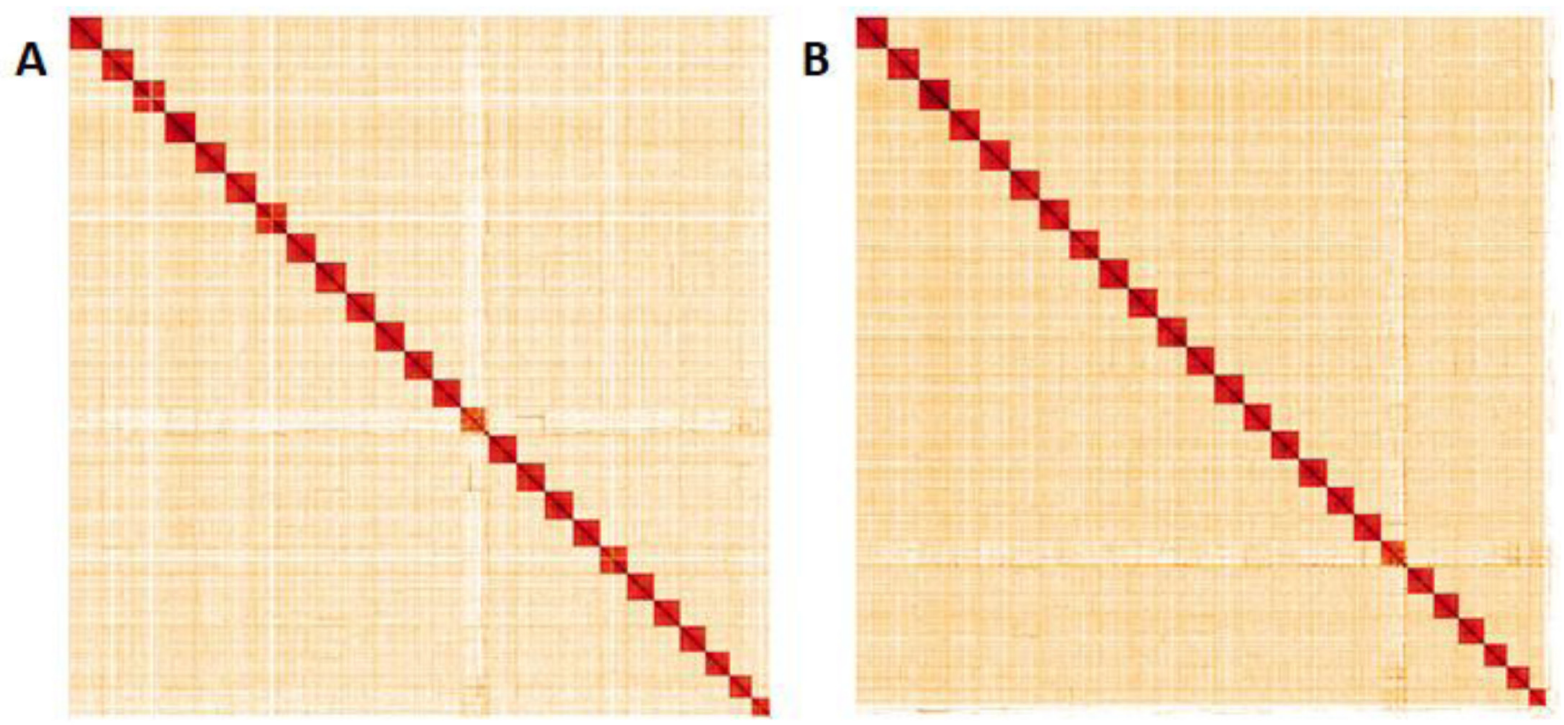
Genome assembly of *Pieris napi*, ilPieNapi4.1 (male, A) and ilPieNapi1.1 (female, B): Hi-C contact map. Hi-C contact map of the ilPieNapi4.1 and ilPieNapi1.1 assemblies, visualised in HiGlass. Chromosomes are given in order of size from left to right and top to bottom.

**Table 2.** Chromosomal pseudomolecules in the genome assembly of *Pieris napi*, ilPieNapi4.1 (male) and ilPieNapi1.1 (female).

Chromosome	Male			Female		
	Size (Mb)	GC%	INSDC accession	Size (Mb)	GC%	INSDC accession
1	15.06	33.7	HG993162.1	14.82	33.7	FR997694.1
2	14.34	33.7	HG993163.1	14.22	33.7	FR997695.1
3	13.84	33.7	HG993166.1	13.69	33.8	FR997697.1
4	13.51	33.9	HG993169.1	13.66	33.9	FR997698.1
5	13.50	33.8	HG993170.1	13.54	33.8	FR997699.1
6	13.82	33.8	HG993167.1	13.46	33.7	FR997700.1
7	14.17	34.2	HG993164.1	13.4	33.7	FR997701.1
8	13.20	33.6	HG993172.1	13.34	33.6	FR997702.1
9	13.39	33.5	HG993171.1	13.29	33.5	FR997703.1
10	13.69	33.8	HG993168.1	13.26	33.7	FR997704.1
11	12.95	33.9	HG993174.1	13.07	33.9	FR997705.1
12	12.84	33.8	HG993176.1	12.99	33.9	FR997706.1
13	12.73	34	HG993177.1	12.73	33.9	FR997707.1
14	12.98	34	HG993173.1	12.71	33.8	FR997708.1
15	12.51	34.2	HG993178.1	12.55	34.2	FR997709.1
16	12.49	33.6	HG993179.1	12.52	33.7	FR997710.1
17	12.22	33.9	HG993181.1	12.2	33.7	FR997711.1
18	12.87	35.8	HG993175.1	12.12	36	FR997712.1
19	12.39	34.3	HG993180.1	11.73	33.9	FR997713.1
20	11.57	34.2	HG993183.1	11.52	34.1	FR997714.1
21	11.75	33.8	HG993182.1	11.48	33.7	FR997715.1
22	10.92	34.2	HG993184.1	10.7	34	FR997716.1
23	10.42	34.6	HG993185.1	10.19	34.4	FR997717.1
24	7.95	35.4	HG993186.1	7.39	34.9	FR997718.1
W	-	-	-	2.68	35.2	FR997719.1
Z	14.07	34	HG993165.1	13.95	33.9	FR997696.1
MT	0.02	19.9	HG993187.1	0.02	19.6	FR997720.1
Unplaced	0.80	41	-	1.99	35.4	-

## Gene annotation

The Ensembl gene annotation system ( Aken *et al.*, 2016) was used to generate annotation for the male *Pieris napi* assembly ilPieNapi4.1 (GCA_905231885.1, see https://rapid.ensembl.org/Pieris_napi_GCA_905231885.1; Table 1). The annotation was created primarily through alignment of transcriptomic data to the genome, with gap filling via protein to-genome alignments of a select set of proteins from UniProt ( UniProt Consortium, 2019) and OrthoDB ( Kriventseva *et al.*, 2008). Prediction tools, CPC2 ( Kang *et al.*, 2017) and RNAsamba ( Camargo *et al.*, 2020), were used to aid determination of protein coding genes.

## Methods

### Sample acquisition and nucleic acid extraction

Three male (ilPieNapi4, genome assembly; ilPieNapi5, Hi-C; ilPieNapi6, RNAseq) and one female (ilPieNapi1, genome assembly) *P. napi* specimens were collected from Carrifran Wildwood, Scotland (latitude 55.400132, longitude -3.3352) by Konrad Lohse, University of Edinburgh, who also identified the specimens. A second female *P. napi* specimen (ilPieNapi9, RNA-Seq) was collected by Alex Hayward, University of Exeter, who also identified the specimen. All specimens were caught with a handnet and were snap-frozen in liquid nitrogen.

DNA was extracted from the whole organisms of ilPieNapi1 and ilPieNapi4 at the Wellcome Sanger Institute (WSI) Scientific Operations core from the whole organism using the Qiagen MagAttract HMW DNA kit, according to the manufacturer’s instructions. RNA (from the whole organisms of ilPieNpi6 and ilPieNapi9) was extracted in the Tree of Life Laboratory at the WSI using TRIzol, according to the manufacturer’s instructions. RNA was then eluted in 50 μl RNAse-free water and its concentration RNA assessed using a Nanodrop spectrophotometer and Qubit Fluorometer using the Qubit RNA Broad-Range (BR) Assay kit. Analysis of the integrity of the RNA was done using Agilent RNA 6000 Pico Kit and Eukaryotic Total RNA assay.

### Sequencing

Pacific Biosciences HiFi circular consensus and 10X Genomics read cloud DNA sequencing libraries were constructed according to the manufacturers’ instructions. Poly(A) RNA-Seq libraries were constructed using the NEB Ultra II RNA Library Prep kit. DNA and RNA sequencing was performed by the Scientific Operations core at the WSI on Pacific Biosciences SEQUEL II (HiFi), Illumina HiSeq X (10X) and Illumina HiSeq 4000 (RNA-Seq) instruments. Hi-C data were generated from the whole organism of ilPieNapi5 using the Arima v1.0 kit and sequenced on HiSeq X.

### Genome assembly

Assembly of both genomes was carried out with HiCanu ( Nurk *et al.*, 2020). Haplotypic duplication was identified and removed with purge_dups ( Guan *et al.*, 2020). One round of polishing was performed by aligning 10X Genomics read data to the assembly with longranger align, calling variants with freebayes ( Garrison & Marth, 2012). The assemblies were then scaffolded with Hi-C data ( Rao *et al.*, 2014) using SALSA2 ( Ghurye *et al.*, 2019). The assemblies were checked for contamination and corrected using the gEVAL system ( Chow *et al.*, 2016) as described previously ( Howe *et al.*, 2021). Manual curation was performed using gEVAL, HiGlass ( Kerpedjiev *et al.*, 2018) and Pretext. The mitochondrial genomes were assembled using MitoHiFi ( Uliano-Silva *et al.*, 2021). The genomes were analysed and BUSCO scores generated within the BlobToolKit environment ( Challis *et al.*, 2020). Table 3 contains a list of all software tool versions used, where appropriate.

**Table 3.** Software tools used.

Software tool	Version	Source
HiCanu	2.1	Nurk *et al.*, 2020
purge_dups	1.2.3	Guan *et al.*, 2020
SALSA2	2.2	Ghurye *et al.*, 2019
longranger align	2.2.2	https://support.10xgenomics.com/genome-exome/ software/pipelines/latest/advanced/other-pipelines
freebayes	1.3.1-17-gaa2ace8	Garrison & Marth, 2012
MitoHiFi	1.0	Uliano-Silva *et al.*, 2021
gEVAL	N/A	Chow *et al.*, 2016
HiGlass	1.11.6	Kerpedjiev *et al.*, 2018
PretextView	0.1.x	https://github.com/wtsi-hpag/PretextView
BlobToolKit	2.6.2	Challis *et al.*, 2020

### Ethics and compliance issues

The materials that have contributed to this genome note were supplied by a Tree of Life collaborator. The Wellcome Sanger Institute employs a process whereby due diligence is carried out proportionate to the nature of the materials themselves, and the circumstances under which they have been/are to be collected and provided for use. The purpose of this is to address and mitigate any potential legal and/or ethical implications of receipt and use of the materials as part of the research project, and to ensure that in doing so we align with best practice wherever possible.

The overarching areas of consideration are:

- Ethical review of provenance and sourcing of the material;
- Legality of collection, transfer and use (national and international).

Each transfer of samples is undertaken according to a Research Collaboration Agreement or Material Transfer Agreement entered into by the Tree of Life collaborator, Genome Research Limited (operating as the Wellcome Sanger Institute) and in some circumstances other Tree of Life collaborators.

## Data availability

European Nucleotide Archive: Pieris napi (green-veined white). Accession number PRJEB43034; https://identifiers.org/ena.embl/PRJEB43034.

The genome sequences are released openly for reuse. The *P. napi* genome sequencing initiative is part of the Darwin Tree of Life (DToL) project. All raw sequence data and the assembly have been deposited in INSDC databases. The female genome will be annotated using the RNA-Seq data and presented through the Ensembl pipeline at the European Bioinformatics Institute. Raw data and assembly accession identifiers are reported in Table 1.
