# Supplementary material for: The genome sequences of the male and female green-veined white, Pieris napi (Linnaeus, 1758)
Source: Wellcome Open Res. 2021 Oct 26;6:288. [Version 1] doi: 10.12688/wellcomeopenres.17277.1 (PMC9257262; doi:10.12688/wellcomeopenres.17277.1)
Supplement: Peer review history

# Peer review: Reviewer response for version 1

**DOI:** 10.21956/wellcomeopenres.19101.r51345

**Published:** 2022-07-05

**Authors:** Ching-Ho Chang

**Affiliations:** 1 Department of Biology, University of Rochester, Rochester, NY, USA

Lohse *et al.* report genome assemblies of two green-veined white. The high completeness and contiguity indicate that the quality of the genome assemblies are very high. However, I feel that some extra analyses would greatly improve the significance of the study. I also found that the resolution of figures needs to be improved.

 I am mostly questioning what contributes to the difference in the size of the assemblies. The previously published assembly is 349.8 Mb, and the assemblies generated in this study are 320 Mb for the male and 319 Mb for the female. Moreover, although a female has an extra W chromosome, its assembly size is the smallest. The difference can result from strain difference or assembly quality. I hope that the author can compare assemblies by plotting dot plot chromosome-by-chromosome. This way, we will have better ideas about which causes the difference. We can probably see some polymorphic inversions in this way. I’m also wondering why the authors chose male, instead of female, as their reference genome to annotate.

Are sufficient details of methods and materials provided to allow replication by others?

Yes

Is the rationale for creating the dataset(s) clearly described?

Yes

Are the datasets clearly presented in a useable and accessible format?

Yes

Are the protocols appropriate and is the work technically sound?

Yes

Reviewer Expertise:

Genomics

I confirm that I have read this submission and believe that I have an appropriate level of expertise to confirm that it is of an acceptable scientific standard.

# Peer review: Reviewer response for version 1

**DOI:** 10.21956/wellcomeopenres.19101.r46702

**Published:** 2021-11-17

**Authors:** Liuqi Gu

**Affiliations:** 1 Beijing Huayuan Academy of Biotechnology, Beijing, China

Lohse *et al.* here report new chromosome-level genome assemblies of male and female *Pieris napi* butterflies using PacBio, HiC and 10X Gen sequencing data. The methods are clearly documented and the assembly metrics support a high quality assembly. Notably, As part of the Darwin Tree of Life Project, data yielded from this work supplement previously published *P. napi *genome (Hill *et al.*, 2019) and will benefit future research on the biology and ecology of this butterfly. 

 Note that the current assemblies of ~320M is a little smaller than 349.8 Mb reported by Hill *et al.*, 2019, any possible explanation?

 Also, most of the figures are not in production quality with most texts barely legible.

Are sufficient details of methods and materials provided to allow replication by others?

Yes

Is the rationale for creating the dataset(s) clearly described?

Yes

Are the datasets clearly presented in a useable and accessible format?

Yes

Are the protocols appropriate and is the work technically sound?

Yes

Reviewer Expertise:

lepidopteran genomics, gene expression

I confirm that I have read this submission and believe that I have an appropriate level of expertise to confirm that it is of an acceptable scientific standard.
